# Assessing the Potentialities of FORMOSAT-2 Data for Water and Crop Monitoring at Small Regional Scale in South-Eastern France

**DOI:** 10.3390/s8053460

**Published:** 2008-05-23

**Authors:** Dominique Courault, Aline Bsaibes, Emmanuel Kpemlie, Rachid Hadria, Olivier Hagolle, Olivier Marloie, Jean-F. Hanocq, Albert Olioso, Nadine Bertrand, Véronique Desfonds

**Affiliations:** 1 UMR 1114 INRA-UAPV EMMAH, Domaine St Paul, 84914 Avignon, France; E-mails: courault@avignon.inra.fr; absaibes@avignon.inra.fr; ekpemlie@avignon.inra.fr; 2 CNES (DCT/SI/MO) et CESBIO BPI 811, 18 avenue E.Belin, 31401 Toulouse Cedex 9, France

**Keywords:** FORMOSAT-2, crop monitoring, evapotranspiration, LAI, albedo, surface temperature

## Abstract

Water monitoring at the scale of a small agricultural region is a key point to insure a good crop development particularly in South-Eastern France, where extreme climatic conditions result in long dry periods in spring and summer with very sparse precipitation events, corresponding to a crucial period of crop development. Remote sensing with the increasing imagery resolution is a useful tool to provide information on plant water status over various temporal and spatial scales. The current study focussed on assessing the potentialities of FORMOSAT-2 data, characterized by high spatial (8m pixel) and temporal resolutions (1-3 day/time revisit), to improve crop modeling and spatial estimation of the main land properties. Thirty cloud free images were acquired from March to October 2006 over a small region called Crau-Camargue in SE France, while numerous ground measurements were performed simultaneously over various crop types. We have compared two models simulating energy transfers between soil, vegetation and atmosphere: SEBAL and PBLs. Maps of evapotranspiration were analyzed according to the agricultural practices at field scale. These practices were well identified from FORMOSAT-2 images, which provided accurate input surface parameters to the SVAT models.

## 1. Introduction

**Figure 1.**
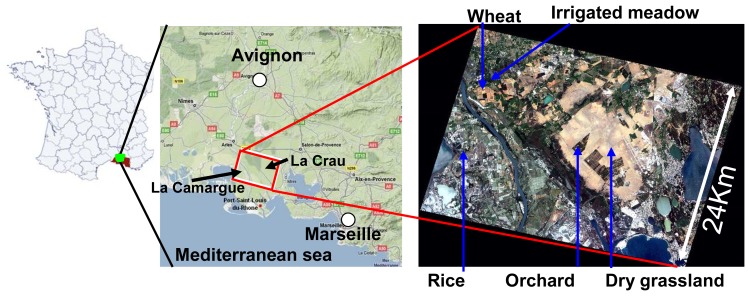
Localisation of the study area (‘Crau-Camargue’ region) and the track of FORMOSAT-2 image, with the main cover types indicated on a true color image.

Describing soil - vegetation - atmosphere transfers is of prime interest for understanding interactions and feedbacks between vegetation and boundary layer, and assessing plant water status [1]. This is gaining importance over these last years in the context of climatic changes, because land use evolution can induce significant modifications of surface fluxes like evapotranspiration, and therefore result in different irrigation strategies. The Crau-Camargue region located in South-Eastern France (Figure 1) is an interesting study case, particularly sensitive to global changes. It is a flat region characterized by highly contrasted humid areas, with a high diversity of crops and agricultural practices. The Crau region is famous for irrigated meadows providing COP (Certified Origin Product) hay exported all over the world. While the Camargue region is known for its rice fields that were initially cultivated for soil salinity remediation purposes [2]. Significant modifications have been noticed these last 10 years. Climatic hazards such as long and dry spring periods and heavy rains in autumn are becoming more frequent. Consequently, the increasing water scarcity during the growing season, in addition to water redistribution for the industrial and urban sectors, has led the authorities to restrict irrigation. Irrigated crops like meadow, which are dominant in the Crau region, were affected by those restrictions. Other crops such as corn, demanding large water quantity during summer period, disappear progressively of the cultural landscape. Moreover, rice plantations in Camargue show large differences in yields (mainly due to the various cultural practices performed in terms of sowing dates, submersion dates after sowing and drying up dates before harvesting [2]). It is therefore important to estimate with accuracy this surface variability, to know the real water need for each field so as to improve crop and water management.

Remote sensing with the increasing imagery resolution is an useful tool to provide such information on plant water status over various temporal and spatial scales [3]. Evapotranspiration (ET) may be estimated from remote sensing data with different approaches: direct methods using thermal infrared *(TIR)* data, indirect estimates using assimilation procedures combining different wavelengths to get various input parameters [23]. However, the use of remote sensing for operational applications still presents several problems. Water and crop monitoring requires a frequent time revisit of satellites and a fine spatial resolution to get accurate information at the field scale. The relatively long time revisit (16 days) for satellites having a high spatial resolution such as ASTER or Landsat make their use for operational applications often unattractive (even if they have thermal spectral bands which are used in numerous ET models). Recent satellites such as FORMOSAT-2 offer both a high spatial and temporal resolution, since they can revisit a same area, every day with a constant viewing angle, with a pixel of 8m (for FORMOSAT in multispectral range or 2m in black and white). This new sensor generation characterized by repetitive acquisitions of high resolution images is very useful for monitoring land surface dynamics. Future missions, such as Venμs [4] or Sentinel2 [42]), having similar characteristics, are thus defined to be part of the GMES program (Global Monitoring for Environment and Security) with larger objectives dealing on the environment surveillance (http://www.gmes.info/). However, these last satellites do not have spectral bands in the thermal range. Several missions (conducted by CNES: Centre National d´Etudes Spatiales, Toulouse 3w/cnes.fr: IRSUTE [56], SEXTET [34]) have studied the interest of *TIR* data acquired at fine spatial and temporal resolution. Actually there is no current satellites which have these specific configurations. Recent works explore future missions to answer to this strong demand (let us mention the MISTIGRI mission conducted by the CNES). While waiting for operational solutions, alternative approaches are proposed combining information at different wavelengths and resolutions [56].

A heavy experimentation took place over the Crau-Camargue region in 2006, including intensive ground measurements on different crop types, in parallel to airborne and satellite data collection. Thirty cloudless FORMOSAT-2 images were acquired from March to October. Thermal data were taken at fine resolution from an airborne thermal camera (3.5m) at few dates during this period (see table 1), while ASTER and Landsat 5 images were recorded at a larger resolution.

The objectives of this study were i) to evaluate the potentialities of FORMOSAT-2 data for mapping the main land properties, and ii) to estimate and better understand the spatial variability of surface fluxes and microclimate over this region, using operative models requiring minimum input data combining multi spectral data acquired at fine spatial resolution. These models are based on data acquired in both the optical and thermal domains. The microclimate is represented here by its main variable: air temperature which is strongly related to land surface thermal exchanges, vegetation functioning, and to the plant growth.

Besides, let us mention that it was the first time that such remote sensing data (FORMOSAT-2) are used for agricultural applications. The interest of the high temporal resolution was particularly important for this study area, where irrigation was applied to different surfaces with a high frequency (every 8-11 days over the meadows). The impact of these various agricultural practices has a great influence on all exchanges between surface and atmosphere.

For this work, we have used different types of models, first to estimate the main biophysical variables characterizing the various crops, and then to simulate surface fluxes and air temperature. Section 2 presents the dataset used. The models based, either on physical or semi-empirical relationships, are briefly described in section 3. The main results are discussed in section 4. Finally a conclusion is made on the potentialities of FORMOSAT-2 data and the future applications.

## 2. Dataset

### 2.1. Ground measurements

The study area (centered at 2.3E 45.89N 4E 47.69N, figure 1) is characterized by various crop types. Five representative fields were chosen for intensive ground measurements: two wheat fields sown in winter at different dates and which evolved into bare soils around the end of June, a meadow field flooded every 11 days, and cut three times per year, a sprinkler irrigated corn field (depending on weather conditions), and a rice plantation, sown in April and then submerged by water during its vegetative period with a fluctuating water level until harvesting in October.

On these five fields, meteorological measurements like rainfall, air temperature and moisture, wind speed, global and atmospheric radiations were recorded from March to October 2006. Values were averaged over a time step of 10 min.

Albedo was measured with albedometers (Kipp & Zonen CM7) with the same time step during all the cultural cycle of the study fields. The Kipp sensors were calibrated to provide estimates of incoming radiation over the whole spectrum for measurements over 300 to 3000 nm spectral band. Incident radiation was measured with a pyranometer located on the center of the experimental area. The footprints of these measurements ranged from 1000 to 3000m^2^. Additionally, surface temperatures were acquired all along the experiment, with KT17 Heimann radiothermometers on three locations (wheat, meadow and rice).

For a few intensive observation periods, surface fluxes were measured during several days. 1D-anenometers (CA27 T Campbell) were set up on the various surfaces, allowing to compute the sensible heat flux (*H*). Soil heat fluxes (*G*) were measured using soil fluxmeters (HFT-3, REBS), put just below the surfaces. Pyradiometers (Q7 REBS) measured net radiation (*Rn*). Finally, the latent heat flux (*LE*) was obtained by the residual method of the energy balance:

(1) LE=Rn−H−G.

For these intensive observation periods, atmospheric profiles were also acquired according to the weather conditions (low winds) for several days with a tethered balloon up to 200 m above the surface.

The main development stages of the various crops were monitored by different observations and measurements. Crop heights (*h*_veg_) were measured to estimate surface roughness (*z*_0m_=0.13 *h*_veg_) [5] needed to simulate fluxes over different fields. Leaf Area Index (*LAI*) was estimated for each field by both planimetry and hemispherical photographies. Comparisons between both methods were performed for some dates and gave satisfactory results. We chose the second method for this study, as it was easier to implement. For this indirect method, we used the CAN-EYE software (http://www.avignon.inra.fr/can_eye/page5.php) developed by Weiss and Baret at INRA Avignon to process the image series (technical report at http://www.avignon.inra.fr/can_eye/page5.php). This software allows to obtain different surface parameters such as *fCover* (vegetation fraction), *FAPAR* (fraction of absorbed photosynthetically active radiation) and the *Effective LAI* (that does not take into account vegetation clumping effect), which are comparable to remote sensing estimations [6]. For each study field, 40 to 60 photographies were taken along transects in cross sectional pattern, according to the surface heterogeneity and the field size; (the temporal sampling was done according to the crop development). Since *LAI* measurements were punctual, temporal interpolation of ground measurements using Koetz et al. [7] model was done in order to compare them to *LAI* estimated from FORMOSAT-2 images (described later). Table 1 summarizes the main measurements performed during the experiment.

### 2.2. Remote sensing data and image processing

Thirty cloud free FORMOSAT-2 images were acquired every 3 to 4 days during 8 months at 10:30 TU from March to October 2006, and with a constant viewing angle of 41° over the Crau-Camargue region (Figure 1). FORMOSAT-2 is a Taiwanese high resolution satellite launched in May 2004. It provides images (distribution by SPOT-IMAGE) with the following features: spatial resolution of 8 m in four spectral bands centred at 488, 555, 650 and 830 nm, with a field of view of 24 km, an orbital cycle of one day, with a constant observation angle (see table 2 in appendix for its main features). This data set was first geolocated, registered, calibrated and the cloud and their shadows were discarded by a CNES-Cesbio team in Toulouse according to the method described in Baillarin et al, [8]. Then the images were corrected from atmospheric effects using the atmospheric correction method developed by Hagolle et al. [9] based on the inversion of an atmospheric radiative transfer model.

Additionally Thermal Infrared (TIR) data were collected at different spatial resolutions using spaceborne (ASTER: 1 image acquired on July 26, LANDSAT 5TM: 8 images for the study period), and airborne (FLIR camera) sensors. As mentioned in introduction, these data are necessary to estimate surface fluxes. The main problem is that we cannot have the same spatial and temporal resolution with the current satellites as FORMOSAT-2 data. Thus airborne thermal images (covering the spectral band: 7.5-13μm) were acquired in the same period (on a time interval of 10min ) as the ASTER passage, at 3000m above the surface, along several transects over the implemented fields, leading to a spatial resolution of 3.5 m with a swath of 1.3 km. Five dates from March to September were analyzed. Atmospheric corrections were performed using MODTRAN model [10] and atmospheric profiles obtained from a tethered balloon combined to outputs of the ECMWF (European Centre for Meduim Range Weather Forecast, 3w.ecmwf.int/index.html ) model for the highest atmospheric levels. The TES (Temperature Emissivity Separation) algorithm [11] was used to retrieve surface radiometric temperature from ASTER data. TES relies on the spectral variability captured from the multispectral brightness temperatures within the 5 ASTER TIR bands at 90m spatial resolution. The estimated precision (compared with the ground measurements) is around 0.5°C for temperature computed from the airborne camera and in the order of 1.5°C for the ASTER data. In this paper, we only focussed on the combination of FORMOSAT and TIR airborne data. Studies are ongoing on comparison between ASTER and airborne TIR data.

## 3. Methods

### 3.1. Models for assessing surface fluxes and microclimate

Two models simulating the transfers between soil-vegetation and atmosphere were used: the spatially distributed energy balance model SEBAL [12] (modified version by [14]) and the ‘Planetary Boundary Layer Model (described in [13]). They were chosen because they rely on different assumptions and need only few input parameters easily computed from remote sensing data. Both are based on a single source approach which consists in considering only one surface resistance for the combined soil-canopy system. This approach assumes that all the surfaces can be represented by one effective value of temperature and humidity [46]. For more complex canopies, for example sunflower or tiger bush, the two source modeling schemes propose two set of resistances to reproduce the turbulent and radiative exchanges distinguishing soil and vegetation components within the low atmosphere [47]. Even if this last approach seems to be more realistic, many authors have shown that a simple but correctly calibrated single source model gave satisfactory results for describing the energy balance compared to ill parameterized dual source models [48].

SEBAL was designed to avoid the use of micrometeorological measurements, by exploiting the information contained in the spatial variability captured from solar and thermal remotely sensed images. Its main characteristic relies on the determination of wet and dry surfaces on the study area. A spatial analysis is made on the relationship between albedo (*a*) and surface temperature (*T*_s_) to define a threshold, separating evaporative and dry areas. SEBAL calculates the energy partitioning combining physical parameterization and empirical relationships with minimum ground data, and computes wind speed and air temperature (*T*_a_). The latent heat flux (*or evapotranspiration*) is computed at the satellite acquisition time, as the residual of the energy balance. A detailed description can been found in [12]. The main advantages of this model are that i) the key points have been validated by Jacob et al., [14]; the model was used with success for various applications [15,16, 53], and ii) it is easy to implement and not expensive in computing time.

The second model used to estimate surface fluxes and temperatures variations over the Crau-Camargue, was the Brunet et al. model [13], that we called here ‘PBLs’. It was initially developped over a homogeneous surface on a daily time scale. This land-surface model consists in coupling the simple soil-vegetation-atmosphere transfer model based on the Penman-Monteith equations, and the planetary boundary layer (*PBL*) model of Tennekes and Driedonks [18]. The surface scheme is based on the resolution of the energy budget. The canopy is treated as a single big leaf where the complexity of vegetation-atmosphere interactions were reduced to a key parameter, the surface resistance (*R*_s_) computed with the same parametrisation used in the ISBA model described by Jacquemin and Noilhan [19]. The formulation for *R*_s_ (derived from the Jarvis approach) depends upon both atmospheric factors and available water in the soil (equation 2)

(2) $$R_{s}=\frac{R_{s\min}}{f_{1}f_{2}f_{3}f_{4}\text{LAI}}$$

Where *R_smin_* is the minimum stomatal resistance, *f_1-4_* are limiting factors varying from 0 to 1. They depend on atmospheric and soil moisture conditions. They are described in detail in [19, 21]).

PBLs takes into account the feedbacks between surface and atmosphere via the following simple equation:

(3) $$\frac{\partial X}{\partial t}=\frac{1}{h}(F_{XS}-F_{XH})+CF_{x}$$

Where *t* is time, *X* is air potential temperature (*T*_p_) or air moisture (*q*), *CF*_x_ is a term due to the Coriolis force and *F*_XS_ and *F*_XH_ are the vertical fluxes at the bottom and the top of the mixed layer. *F*_XS_ correspond to the convective fluxes computed by the big leaf surface model.

**Figure 2.**
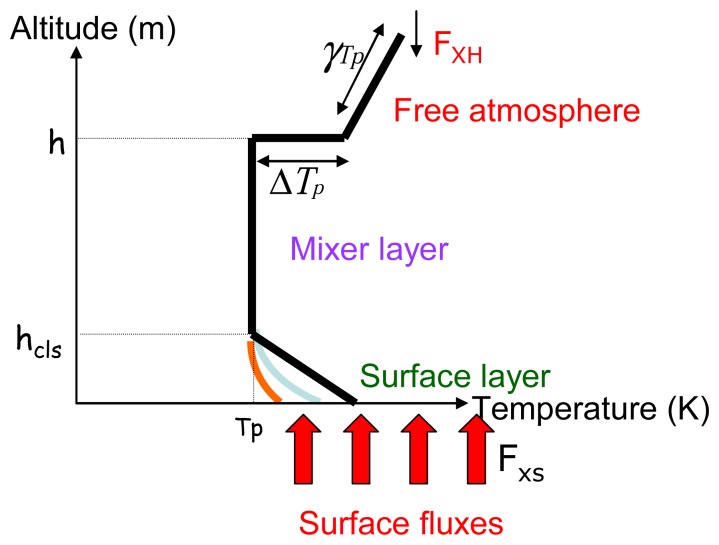
Simplified description of the atmospheric profile adopted in the PBLs model (symbols defined in the text).

As the landscape is heterogeneous, we have modified the surface scheme, in order to take into account the surface variability, introducing a ‘tile approach’ to compute the *Fx* term [20, 50]. The method consists in averaging the surface fluxes computed over each vegetation class, weighted by their percent of surface occupation (α) according to equation 4 (Figure 2).

(4) $$\sum_{i}^{n}\alpha_{i}R_{ni}=\sum_{i}^{n}\alpha_{i}[H_{i}+LE_{i}+G_{i}]$$

This tile approach requests spatially distributed variables derived from remote sensing data, described in the next section. Other variables were also requested by the model, like the initialising variables for the atmosphere, the daily evolution of the global radiation and windspeed. Temperature and humidity profiles were initiated using radio-sounding measurements. Seven characteristic points define these profiles : *T*_p,_ the potential temperature, (*q_s_*: the specific humidity) at the surface boundary layer height (*h_CLS_*), *γ_Tp_* (and *γ_qs_* respectively) the slopes of temperature (and humidity) evolution in the free atmosphere, and *ΔT_p_* (*Δq_s_*) the jump of temperature (or humidity) between free and mixed atmosphere (figure 2). The model computes all the fluxes of the energy balance in addition to surface temperature, air temperature (*T*_a_) and air moisture, (*q*) at each time step, all along the day.

### 3.2. Models used for estimation of biophysical variables from FORMOSAT-2 data

High resolution FORMOSAT-2 images were used to spatially estimate the input parameters necessary to SEBAL and PBLs. Albedo (*a*), roughness (*z_0m_*), emissivity (*ɛ*) were common to the two models. Some parameters were derived from a landuse map, taking into account both the ground measurements and the expert knowledge on the crops. Others were computed using semi-empirical models combining reflectances in different spectral ranges as summarized in table 3.

First a land use classification was done, based on a maximum likelihood supervised classification from images acquired at 5 dates, chosen at the main stages of crop development. Sixteen classes were identified. Those classes included vegetation covers as well as free water surfaces and bare soil. This map has been then improved introducing information about the agricultural practices applied to some crops.

**Figure 3.**
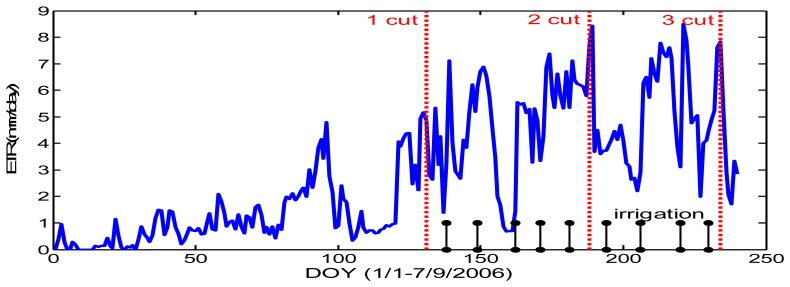
Simulation of evapotranspiration performed with a crop model (STICS) over the irrigated meadow flooded every 11 days and cut 3 times from 1^st^ January to 31 August 2006.

Indeed, the identification of the main cultural practices performed at the field scale, such as irrigation, meadow cut, harvest, plowing, sowing date, is crucial for an accurate determination of the main surface parameters. For example, the roughness map was deduced from the Brutsaert´relationship (*z*_0_=*0.13h_veg_*), *h_veg_* being the mean height measured for each vegetation cover. The crop heights were derived from the landuse map and expert knowledge. All the meadows were not cut at the same date. According to the cut date and irrigation frequency, we have observed from our ground measurements that crop height varied from 0.05 m to 0.6 m, leading to evapotranspiration values varying from 1 to 7 mm per day. Here two factors have an impact on the decrease of evapotranspiration; irrigation and LAI decrease due to the cut. Simulations performed over irrigated meadow with a crop model (STICS, described in [51]) clearly highlighted this large difference of evapotranspiration due to these agricultural practices (figure 3). It is therefore important to know with accuracy the occurrence of such main practices.

**Figure 4.**
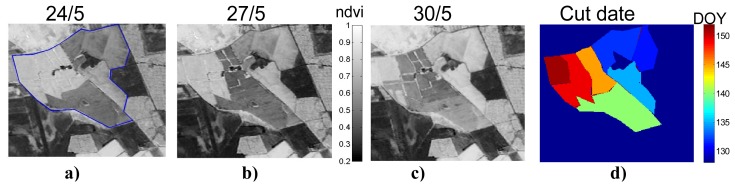
NDVI computed for 3 consecutive dates in April a)24/5 b)27/5, c)30/5/2006 from FORMOSAT images zoomed on irrigated meadows allowing to detect d) the date of the grass cut for the different fields.(see appendix for NDVI definition).

The high spatial and temporal resolution of the FORMOSAT-2 images allowed to track these surface modifications as it is displayed on figure 4. Three successive NDVI images taken at the end of April, zoomed over irrigated meadow fields, showed clearly the freshly cut fields which are associated to large drops in NDVI. From the analysis of these temporal variations, a map of the cut dates was done for the irrigated meadows (fig 4d).

This information has been also used to derive maps of some parameters occurring in the formulation of the surface resistance (*R*_s_) applied in the PBLs model (eq 2). Values for minimum resistances: (*R*_smin_) were affected for each crop class according to the landuse map accounting cultural practices performed (for example *R_smin_* was lower for irrigated fields than for dry crops).

Among the four limiting factors appearing in equation 2, the most important is *f*_2_ which represents the root zone available water fraction. It varies between 0 (completely dry) and 1 (saturated). It is often difficult to estimate with accuracy because it varies a lot spatially at regional scale and all along a cultural cycle according to crop development stages and for different practices (such as irrigation). Numerous studies have shown the relationships between surface temperature (*Ts*) and plant water status [22, 23]. In SEBAL, *Ts* is an input data that indirectly informs on the spatial variability of soil moisture at regional scale. Concerning PBLs, *Ts* is a model output, and two approaches exist to estimate *f*_2_: either by forcing the model by a *f*_2_ map as accurate as possible, deduced from various observations, or by estimating *f*_2_ through assimilation methods [24]. In a first stage, we have chosen to force PBLs with a *f*_2_ map obtained from remote sensing data and observations. The second approach based on an assimilation procedure to retrieve *f2* is currently under study [20]. For this study, *f*_2_ was deduced from the analysis of the thermal images acquired at fine resolution with the airborne camera, combined with the FORMOSAT image acquired at the same date. We have assumed a negative linear correlation between *T*_s_ and *f*_2_ with minimum *T*_s_ corresponding to a maximum *f*_2_ and inversely, a maximum *T*_s_ corresponding to a minimum *f*_2_ (eq 5).

(5) $${\text{f}}_{2}=\frac{\left({\text{f}}_{2_{\max}}-{\text{f}}_{2_{\min}}).({\text{T}}_{\text{s}}-{\text{T}}_{{\text{s}}_{\min}}\right)}{\left({\text{T}}_{{\text{s}}_{\max}}-{\text{T}}_{\text{s}\min}\right)}+{\text{f}}_{2_{\max}}$$

**Figure 5.**
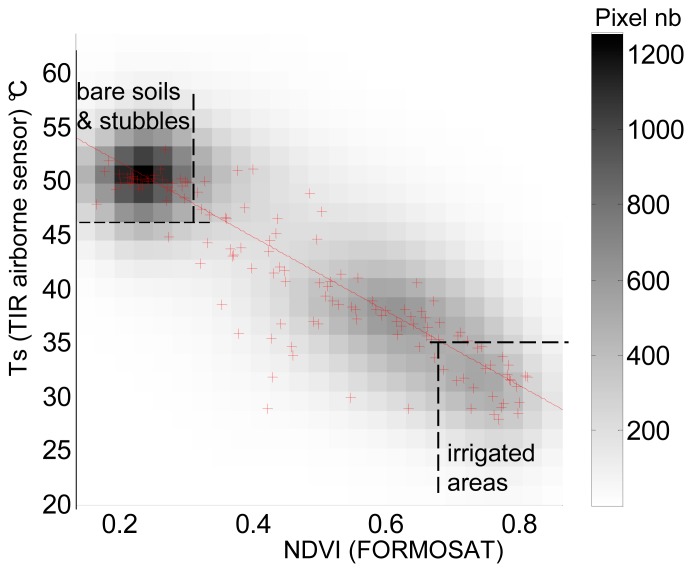
Relationship obtained over a small area covering the measurement fields (5×5km), between NDVI computed from FORMOSAT data and Ts measured from the TIR airborne camera on July 26 at 11:30TU.(the red crosses correspond to NDVI averaged over an class interval of 0.002).

In order to define these extreme values for the studied day, we have analysed the relationship between *Ts* and *NDVI* (figure 5). From a simple thresholding on *Ts* and *NDV*I values, we could well separate the very wet areas corresponding to swamps and meadows that have been flooded (*NDVI*>0.7 and *Ts* < 35°C, for which *f*_2_ will be maximum) from very dry areas corresponding to bare soil or wheat stubbles (*NDVI*<0.3 and *Ts* >47°C, where *f*_2_ is at minimum). The maximum and minimum measured surface temperatures, *T*_s max_ and *T*_s min_, of these extreme surfaces were of 59.8°C and 18.8°C respectively. The estimation of the extremes of *f2* resulted in several PBLs runs, made with different values of *f2*. We have chosen the values which gave the best estimations of sensible heat fluxes compared to our ground measurements. *f*_2 max_ and *f*_2 min_ were therefore set to 0.7 and 0.3 respectively. We noticed that these extremes corresponded to the threshold values of *NDVI* defined to separate irrigated areas from very dry lands. The other dates presented in table 1 are currently under study in order to see if the same relationships can be found for different surface and atmospheric conditions.

For *LAI* estimation, among the various models proposed in the literature (see reviews made by [25]), we chose the simple relation initially proposed by Asrar et al. [26], and widely used for various applications [27].

(6) $$\text{LAI}=-\left(\frac{1}{K_{\text{LAI}}}\right).\text{ln}\left(\frac{\text{NDVI}-{\text{NDVI}}_{\infty }}{{\text{NDVI}}_{s}-{\text{NDVI}}_{\infty }}\right)$$

where *NDVI*_∞_ is the asymptotic value of *NDVI* when *LAI* tends towards a maximum value, that was practically around 7 in our study case, *NDVI*_s_ is the bare soil *NDVI* value and *K*_LAI_ is the extinction coefficient. Those parameters were fitted to measured data using an optimisation approach based on the simplex method, which aimed at minimizing the Root Mean Square Error (*RMSE*) between measured and calculated *LAI*. A ‘leave-one-out’ cross-validation re-sampling method described briefly in appendix [43, 45] was applied to estimate the performance of the approach.

Emissivity maps were then estimated from LAI maps, according to the model proposed by François et al, [28]. Values varied between 0.95 and 0.98.

Various models have also been proposed in the literature to estimate albedo. They vary from simple techniques based on multi linear regressions using bidirectional reflectances acquired in the Visible (VIS) and Near Infrared (*NIR*) ranges [29], to more complex models using radiative transfer models such as SAIL [30] and inversion procedures [31]. Here again, we have chosen the simplest model based on a linear combination of VIS and NIR reflectances (*ρ_vis_*, *ρ_nir_*). As no coefficient set for linear combination of wavebands was yet defined for FORMOSAT-2, we have adjusted a new coefficients set from our data set. Different regression models were tested with several spectral band combinations. Only positive, significant bands were kept and the sum of the coefficients was verified to be almost equal to one as suggested by Jacob et al. [32]. Finally, the best result was obtained with only two bands in the red and near infrared ranges shown in equation 7,

(7) $$a=0.645\rho_{\text{red}}+0.382\rho_{\text{NIR}}$$

Let us mention that different methodologies for assessing LAI and albedo (not described in this paper) have been compared using the same data set [paper submitted 52]. We have chosen in this study only the simplest methods which gave the better results.

## 4. Results - Discussion

### 4.1. Validation of biophysical variables

**Figure 6.**
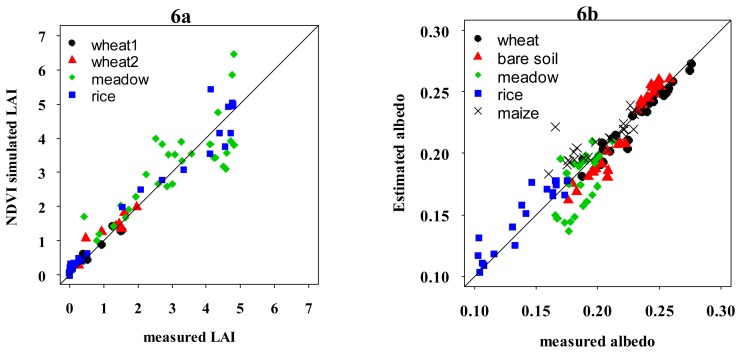
Comparison between **a)** estimated *LAI* based on *NDVI* method and measured using hemispheric photographies and **b)** estimated and measured albedos over the different field from March to October 2006.

The performance criteria (defined in appendix) obtained for albedo estimation, (*RMSEr* = 7.07% and *RSME_A_*=0.014) were very acceptable since they were comparable, on one hand, to the precision obtained by Weiss and Baret [33], and Jacob et al. [32] while estimating shortwave albedo, and, on the other hand, to the albedometer precision which was around 5%. Note that those results were very satisfactory (fig 6b) despite the fact that the seasonal variations of the solar zenith and azimuth angles were neglected. However, we can notice a slight dispersion for meadow and rice which have specific cultural practices (rice always submergred by water, and meadow flooded each 11 days). Let us mention that these relationships must be validated over different vegetation covers in order to evaluate their extrapolation capacities.

**Figure 7.**
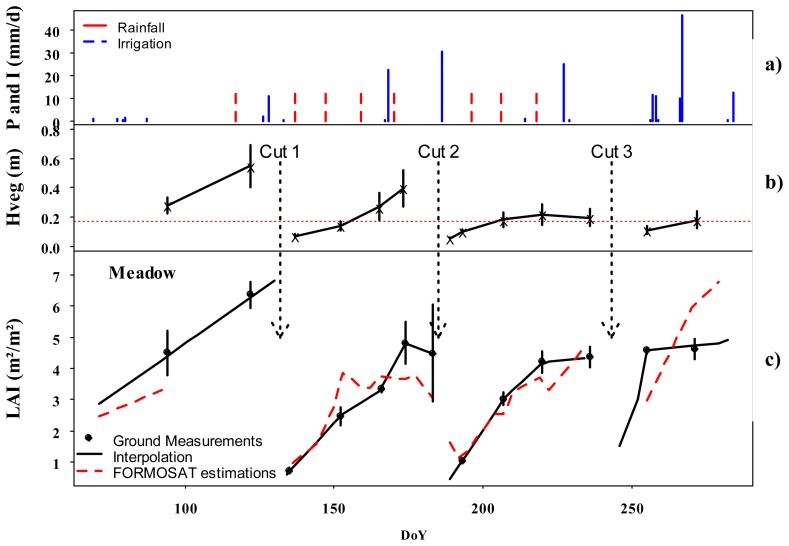
**a)** Rainfall and irrigation events over the study period. **b)** Measured vegetation height with vertical bars corresponding to the 95% confidence interval of measurements. **c)** Comparison between estimated and measured LAI of irrigated meadow over the study period.

For *LAI* estimation, the best fitted values found for *NDVI*_∞_, *NDVI*_s_, and *K_LAI_* were respectively 0.9, 0.1, and 0.70, with a RMSE_R_ of 26.8%. The results compared to ground measurements were globally satisfactory as displayed in figure 6a. Those values were very comparable to those obtained in other studies [27, 35]. However, we can notice more dispersion for irrigated meadows at high *LAI* values. Discrepancies between simulations and measurements for high values of LAI (>4) hare already been observed by several authors [17, 6]. In order to better understand this dispersion, we have analyzed the temporal variation of *LAI*. Figure 7 displays a comparison between the dynamics of estimated and measured meadow *LAI*, the evolution of vegetation height, *h_veg_*, and the main cultural practices performed over the study period (3 cuts and irrigation every 11 days approximately). We can first notice that the 3 cuts, represented by vertical arrows on the figure, are well identified by *LAI* measurements and FORMOSAT data. We saw that simulations gave better results if they were only compared to the measurements with their confidence intervals than to interpolated data. The choice of the linear interpolation of *LAI* appears indeed questionable. Other empirical models used classically to interpolate *LAI* data such as those proposed by [53] can be applied to short period for the meadow between each cut. However such empirical models do not take into account small variations due to various factors such as water stress which can affect *LAI* for small periods between two irrigation events.

Finally, we can conclude that single multidate multicrop relationships empirical approaches yield accurate estimates of *LAI* and albedo from the limited information provided by FORMOSAT-2 sampling (three wave bands and one direction).

### 4.2. Validation of Surface fluxes

Spatial variations of the main energy fluxes and air temperatures were simulated by both models: SEBAL and PBLs for the July 26 2006. Comparisons were made between simulations and measurements accounting for the footprint of the integrated flux measurements. The footprint was computed based on the analytical solution of the diffusion equation of [36].

**Figure 9.**
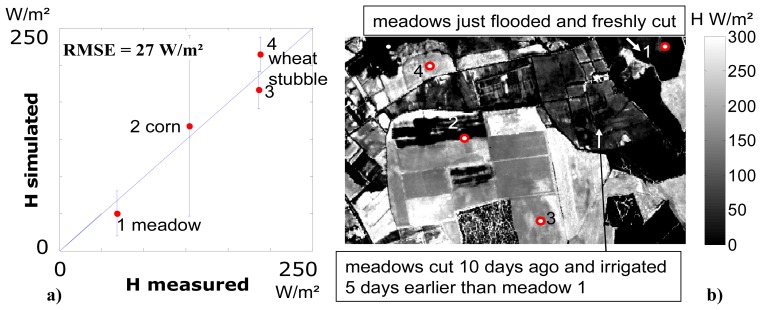
Sensible heat flux computed with the SEBAL model for the July 26 2006 at 11:30 TU, **a)** compared to the measurements performed over various fields, **b)** over a small area extracted from the FORMOSAT and FLIR images.

In general, there was reasonable agreement between sensible flux outputs from both models versus ground measurements (figure 7). The RMSE were respectively of 27 W/m^2^ for SEBAL estimations and of 30 W/m^2^ for the PBLs estimations. Let us mention here that these values were computed, for SEBAL, between instantaneous simulations obtained at 11:30 and measurements performed at the same time (see figure 9). While RMSE computed for PBLs takes into account all values simulated from 6:00 to 18:00 TU compared to the measurement averaged at the same time step. The instantaneous RMSE for PBLs at the time than SEBAL was of 31.7W/m^2^. These errors are acceptable since they were comparable to those found in the literature [1, 23].

The simulated surface fluxes showed large spatial variations due to differences in soil moisture and surface roughness, which were highly dependant on cultural practices performed on each field, as it is shown in figure 9. Irrigation appeared, as expected, as the factor explaining the greatest spatial variation. Irrigated meadows showed the lowest values for sensible heat fluxes (figure 9a), while bare soils or wheat stubbles, which were very dry at this date, had the highest values of sensible heat flux (250 W/m^2^). On Figure 9b we observed different H values for meadows due to differences in irrigation and cut dates.

The large confidence intervals observed for the corn field (fig 9a) were essentially due to the surface heterogeneity. Indeed, this field was sown relatively late on May 5^th^ and was intermittently irrigated by sprinklers depending on weather conditions. The soil was very stony at some locations, with a low water reserve, that explained the bad development of this crop for 2006. The measurement representativeness was therefore arguable over this corn field.

### 4.3. Air temperature estimations

**Figure 10.**
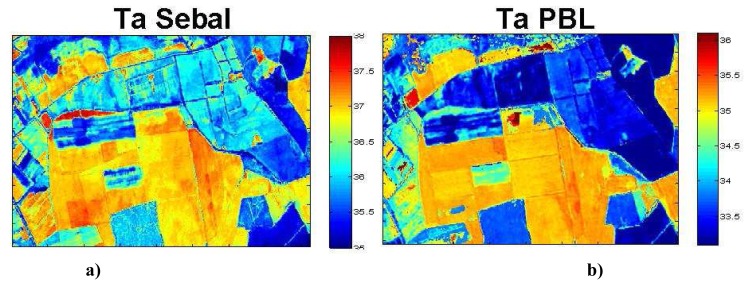
Air temperature estimated at 2m above the surface **a)** from SEBAL model and **b)** PBLs model for the 26 July at 11:30TU.

Figure 10 shows the air temperature maps obtained with SEBAL and PBLs at 11:30TU for the 26^th^ of July. Both models clearly simulated a difference of 3°C between wet and dry areas, which was in the order of the difference observed from the meteorological measurements performed on the various fields and in the range of order of other studies in the same area [55]. However, we noticed that SEBAL gave higher values than PBLs and overestimated by about 2°C when compared to the ground measured temperatures. These results suggest that some of the simplifying assumptions in SEBAL may not be strictly applicable over a wide range of conditions present within this region, in particular, the linear relationship between surface air temperature difference and Ts. Numerous papers have also discussed the problem linked to the choice of the albedo threshold to separate wet and dry area, and its influence on this linear relationship to retrieve above canopy air temperature [1, 37, 54].

## 5. Summary-Conclusion

This study focussed on assessing the potentialities of FORMOSAT-2 data for water and crop monitoring at regional scale. We have shown that the high temporal and spatial resolutions of these new remote sensing data allowed providing accurate surface parameters that lead to satisfactory flux simulations when used as input data in both land-surface models tested. Indeed, identification of each crop type associated with its main cultural practices is possible (such as irrigation and cut date of meadow for example). Roughness map can then be elaborated with more precision. The main surface parameters characterizing the vegetation development such as *LAI*, or the surface radiative properties such as albedo can be derived from these data using simple methodologies easy to implement everywhere. The found relationships were acquired under the specific geometrical configuration of the site, i.e. under 41° zenith view angle and solar zenith angles ranging from 25° up to 45°. Application to other conditions may require adaptations, either using BRDF models if well calibrated over the surfaces investigated, or replication of the whole experimental process under these new conditions. Alternative approaches based on radiative transfer model inversion were not yet applied from this study, and should require further efforts. However, the fact that single multidate multicrop relationships based on empirical approaches yield accurate estimates of *LAI* and albedo from the limited information provided by FORMOSAT-2 sampling (three wave bands and one direction) indicates that this might be possible under well defined prior information.

Preliminary simulations using two different land surface models (SEBAL and PBLs) were performed using input parameters derived from FORMOSAT-2 data, to compute surface fluxes and air temperature above canopy. The results obtained for flux estimations were satisfactory for both models with a slight overestimation for microclimate variables simulated with SEBAL due to simplified assumptions used in this model. Both models were based on surface energy balance with a single source approach. This study has shown that with minimum ancillary information, simple models could be used for water management with quite acceptable results [53]. However, it would be required to have remote sensing data at high spatial and temporal resolution. FORMOSAT-2 provides images every day in four spectral bands in the visible and near infrared domains. Thermal data are also necessary at fine resolution. Currently, there are no satellites which provide similar temporal and spatial resolution such as FORMOSAT-2 in this spectral range. MODIS (EOS) or AVHRR (from NOAA meteorological satellites) deliver thermal data on a daily basis but with a coarse spatial resolution of 1km. A higher resolution was achieved by Landsat (TM: 120m, ETM: 60m), and ASTER (90m) but the time revisit is low (16 days), and do not allow to detect for example meadow irrigation occurring every 11 days in our region. There is currently a strong demand from the scientific community for having thermal sensors with finer resolution, such as the former European SPECTRA mission which yielded the Chinese SPECTLA mission (Menenti, 2005 personal communication), or future MISTIGRI mission currently in study by CNES [34]. Meanwhile, recent works have explored the possibility to use simultaneously various spatial resolutions [39] Different methodologies have been proposed to disaggregate large pixels of *T*_s_ to estimate subpixel *T*_s_ combining various information at different wavelengths [40, 41].

A next step for the current study will be to analyse this point which requires more investigations in the future for operational applications, in comparing ASTER and airborne data acquired over the same area.

## Figures and Tables

**Figure 8.**
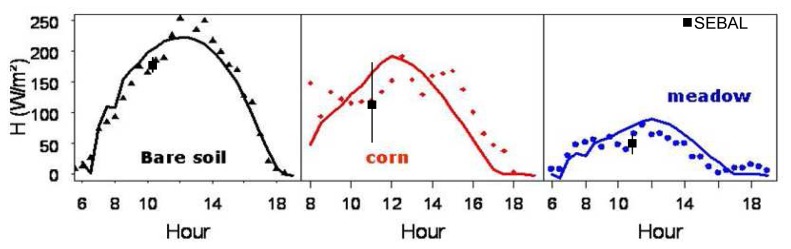
Comparisons between sensible heat fluxes estimated for the main crops with PBLs model (bold line) and field data (points) acquired with 1D anenometers. (SEBAL estimations : black square).

**Table 1.** Main measurements performed on studied fields (Ts : surface temperature - KT 17 Heimann, Ta: air temperature - thermistor 107 Campbell, q: humidity - Vaisala capacity probe: HPMP35D, u: wind-speed - anemometer A100L2).

*Measurements*	*Wheat 1 then stubbes*	*Wheat 2 then bare soil*	*Irrigated meadow*	*corn*	*Rice*

*Micrometeorology (Ta,q,u) + Albedo*	*Continuous from 15/3 to 30/9*	*Continuous from 15/3 to 30/9*	*Continuous from 15/3 to 30/9*	*Continuous from 5/5 to 30/9*	*Continuous from 27/4 to 20/10*
*Ts*	*idem*	*/*	*idem*	*/*	*idem*
*Surface Fluxes*	*8* × *(2-4) days*	*/*	*8* × *(2-4) days*	*/*	*8* × *(2-4) days*
*Crop height*	*6 dates*	*6 dates*	*13 dates*	*5 dates*	*7 dates*
*LAI*	*6 dates*	*6 dates*	*13 dates*	*5 dates*	*7 dates*
*Remote sensing*	*30 FORMOSAT*	*30 FORMOSAT*	*30 FORMOSAT*	*30 FORMOSAT*	*30 FORMOSAT*
*Airborne*	*5 flights*	*5 flights*	*5 flights*	*5 flights*	*5 flights*
*Atmospheric*	*5 dates*	*5 dates*	*5 dates*	*5 dates*	*5 dates*
*Profiles*					

**Table 3.** Input parameters for both models SEBAL and PBLs derived from remote sensing data. (f: empirical function, or semi empirical models described in the next paragraph).

Spatialized inputs	SEBAL	PBLs

Common for both models	Albedo= f(FORMOSAT)Roughness=f(landuse map, FORMOSAT)Emissivity= f(FORMOSAT)	

For each model	Ts=f(FLIR) NDVI	f2=f(FORMOSAT,FLIR) LAI=f(FORMO SAT) +params linked to surface resistance (idem z0)

## Appendix

**Table:** **FORMOSAT2 features** (from http://www.spotimage.fr/web/en/979-formosat-2-imagery-features.php)

Products	Black &White : 2-m
	Colour 2-m (merge)
	Multispectral (R, G, B, NIR): 8-m
	Bundle (separate Pan and MS images)

Spectral bands	P : 0,45 – 0,90 μm (Panchromatic)
	B1 : 0,45 – 0,52 μm (Blue)
	B2 : 0,52 – 0,60 μm (Green)
	B3 : 0,63 – 0,69 μm (Red)
	B4 : 0,76 – 0,90 μm (Near-infrared)

Sensor footprint	24 km × 24 km

Revisit interval	Daily

Viewing angles	Cross-track and along-track (forward/aft): +/- 45°

Satellite tasking	Yes
	Panchromatic and multispectral images can be acquired at the same time

Image dynamics	8 bits/pixel

Image file size (level 1A without metadata)	MS : 35 Mb
	Pan : 137 Mb

### NDVI definition

NDVI=(reflectance _near infrared_- reflectance _red_)/( reflectance _near infrared_+ reflectance _red_

### Performance criteria

Absolute and relative root mean square error (*RMSE_A_* and *RMSE_R_*) between predicted (*P*) and observed (*O*) values were calculated according the following formula, were *n* is the total number of *i* observations, and 〈*O*〉 the mean value of those observed values

$${\text{RMSE}}_{A}=\sqrt{\frac{\sum_{i=1}^{n}{\left(P_{i}-{Ο}_{i}\right)}^{2}}{n}}$$

$${\text{RMSE}}_{R}=\frac{{\text{RMSE}}_{A}}{\langle O\rangle }$$

### Brief description of the ‘leave-one-out’ cross validation method

A ‘leave-one-out’ cross validation method is a statistical method used for estimating generalization error based on “resampling” [43,44,45]. It a computer intensive method used frequently in applied statistics. The method is based on observed data, and allows robust estimation of sampling variances or standard errors and (asymmetrical) confidence intervals. The fundamental idea of the model-based sampling theory approach to statistical inference is that the data arise as a sample from some conceptual probability distribution, *f*. In practical application, the cross validation means that N separate times (N being, the number of data points in the set), the function approximator is trained on all the data except for one point and a prediction is made for that point. the average error is computed and used to evaluate the model.
